# Development and evaluation of a training program for interpreters in the field of trauma-focused cognitive behavioral therapy

**DOI:** 10.3389/fpsyg.2023.1148690

**Published:** 2023-07-19

**Authors:** Lauritz Rudolf Floribert Müller, Monja Lucia Herold, Johanna Unterhitzenberger, Rita Rosner

**Affiliations:** ^1^Department of Psychology, Catholic University of Eichstätt-Ingolstadt, Eichstätt, Germany; ^2^Department of Child and Adolescent Psychiatry, Centre for Children and Adolescents Inn-Salzach e.V., Altoetting, Germany

**Keywords:** Interpreter, language mediator, mental health, refugee, PTSD, children and adolescents, online training

## Abstract

**Background:**

The treatment of traumatized refugee minors is often challenging because of language barriers. International guidelines, therefore, recommend the use of language mediators. However, there is a scarcity of evaluated training programs that prepare language mediators to translate during psychotherapy developed specifically for this patient group, for instance trauma-focused cognitive behavioral therapy (TF-CBT).

**Methods:**

Based on an extensive literature review and in collaboration with an expert focus group, a one-day TF-CBT-specific online training program was developed for language mediators willing to work with minor refugees, and delivered on nine occasions between November 2020 and June 2021. The participants answered pre- and post-training questions about trauma- and TF-CBT-related knowledge and attitudes relevant to therapy, as well as the perceived usefulness of the training. Bayesian estimation was used to determine pre-post changes.

**Results:**

A total of 129 participants speaking 35 different languages participated in the training program. Analyses revealed 95% highest density intervals not containing the null with respect to knowledge gain (effect size median 0.28) and change in treatment-appropriate attitudes (effect size median 0.31). The participants rated the training as useful.

**Conclusion:**

The TF-CBT-specific training course was successfully carried out. It was likely to disseminate both knowledge gains and a shift toward more treatment-appropriate attitudes. It was perceived as useful by the participants. Given the scarcity of evaluated training programs for language mediators working with minor refugees, the results are promising. The limitations include the lack of both a control group and the verification of the results using an external outcome measure.

## Introduction

Many minor and young refugees have been registered in the German youth welfare system due to the increased intake of refugees in Germany since 2014 (BAMF – Bundesamt für Migration und Flüchtlinge, 2020). Although the number of refugees has since decreased, 31,184 young unaccompanied refugees were still being cared for in child and youth welfare services across Germany in November 2019 (Karpernstein and Nordheim, 2019). Minor refugees very often suffer from traumatic experiences (Müller et al., 2019) and are, therefore, at high risk of developing post-traumatic stress disorder (PTSD) (Reavell and Fazil, 2017; Kien et al., 2019). To treat this mental disorder, the National Institute for Health and Care Excellence (National Institute for Health and Care Excellence, 2021) recommends trauma-focused cognitive behavioral therapy (TF-CBT) (Cohen et al., 2017). This form of psychotherapy has already produced promising initial results in the treatment of minor refugees (Unterhitzenberger et al., 2019).

As the German language skills of minor refugees are often insufficient to undergo therapy, the assistance of interpreters is recommended. Most existing research that examined the effectiveness of interpreter-assisted therapy, did not identify any differences between standard therapy with and without an interpreter (Ardenne et al., 2007; Brune et al., 2011; Lambert and Alhassoon, 2015). On the other hand, Sander et al. (2019) found significantly worse outcomes for psychotherapy when interpreters were included. Various qualitative studies that point out the challenges that therapists and interpreters face in triadic settings may provide some explanations for possibly poorer therapy outcomes when interpreters are present (Miller et al., 2005; Morina et al., 2010; Gartley and Due, 2017; Kießl et al., 2017; Hanft-Robert et al., 2018; Geiling et al., 2021). Ratcliff and Suardi (2006) and Ratcliff and Pereira (2019) showed that therapists’ and interpreters’ expectations regarding the role of the interpreter may diverge which makes the relationship susceptible to rivalries, role confusion, partiality, and dissatisfaction (Morina et al., 2010). Thus, a clear distribution of tasks with transparent role clarification is important when it comes to mastering these challenges. The therapist should take responsibility for leading the conversation and the interpreter should facilitate the conversation between the patient and the therapist (Miller et al., 2005; Morina et al., 2010; Kießl et al., 2017; Hanft-Robert et al., 2018). In addition to role clarification at the beginning of psychotherapy, this should be supported by verbatim translations in the first person (Morina et al., 2010; Kießl et al., 2017; Hanft-Robert et al., 2018). Nevertheless, the role of the interpreter should not be seen merely as a mechanical language mediator (Schouler-Ocak and Aichberger, 2017). The person responsible for the translation influences the conversation by their presence and this person must be able to adopt a therapeutic, non-judgmental, and empathic attitude, and to maintain confidentiality (Miller et al., 2005; Gartley and Due, 2017). Therefore, the interpreter needs to have a basic understanding of the therapeutic approach in order to be able to produce qualified translation in psychotherapy settings (Miller et al., 2005; Bauer and Alegría, 2010; Butow et al., 2012; Becher and Wieling, 2015; Kießl et al., 2017; Hanft-Robert et al., 2018; Fennig and Denov, 2021; Villalobos et al., 2021).

Interpreting in child mental health settings is even more challenging as the interpreter has to cope with child development, appropriate verbal and non-verbal language for minors, and settings including the parents (Rousseau et al., 2011). The systematic review by van Os et al. (2020) stresses the importance of well-trained interpreters, especially for this setting. This has also been stated repeatedly by various other authors (Paone and Malott, 2008; Cecchet and Calabrese, 2011; Searight and Armock, 2013; Hunt and Swartz, 2017). Untrained interpreters may not be able to meet ethical standards, and this can prove harmful for traumatized individuals who have already survived situations of betrayal and disloyalty (Crezee et al., 2013), particularly in the case of minors. The evaluation of training courses developed specifically for interpreters working with minors undergoing therapy and the implementation of training courses in practice are thus crucial to ensuring the effectiveness of this type of psychotherapy (Paone and Malott, 2008; Rousseau et al., 2011).

As only a limited number of certified interpreters are available for many of the languages needed for the psychosocial work with minor refugees in Germany, lay interpreters often take on this task (Hanft-Robert et al., 2018). Both inside and outside Germany, a broad spectrum of training courses is available that prepare interpreters for work as language and cultural mediators or community interpreters, mostly in adult settings. Some of these training programs address aspects of translating in psychotherapy (Schouler-Ocak and Aichberger, 2017; Herold, 2019). However, none of the training programs known to the authors that had been specifically designed for work in psychotherapy, has been evaluated scientifically up to now. In contrast, there are some examples of evaluations of interpreter training programs in other disciplines that follow different approaches (Hasbún Avalos et al., 2013; Federici and Cadwell, 2018; Hale et al., 2019). However, none of these evaluations used pre- and post-tests to systematically measure changes.

Some training programs developed for therapists focusing on TF-CBT for the treatment of PTSD have been evaluated in terms of knowledge gain (Murray, 2017) or attitude change (Sansen et al., 2019) using pre- and post-tests. Murray (2017) based his evaluation on the Kirkpatrick’s “Four Levels of Evaluation” (Kirkpatrick, 1975). This is an established theoretical approach in medical education (Yardley and Dornan, 2012). The extensive evaluation is based on four levels comprising (1) a positive “reaction” by the participants, (2) “learning” measured as an actual knowledge gain, (3) “behavior” change in the specific setting which should lead to (4) the desired outcome “results” (Kirkpatrick, 1975; Alliger and Janak, 1989; Kirkpatrick and Kirkpatrick, 2010). Yardley and Dornan (2012) rightly point out the importance of evaluating the last two levels. However, due to the lack of empirical findings on interpreter trainings in the psychotherapeutic field, exploratory evaluations centered on the first two levels are still helpful as they already provide some initial indication of the effectiveness of a training program.

Theories about attitudes and attitude changes state that cognitive capacities and motivation are required to process new information (Petty and Cacioppo, 1986; Chaiken and Maheswaran, 1994; Dillard and Pfau, 2002; Kruglanski et al., 2006; Bohner and Dickel, 2011; van Lange et al., 2011). Participants who already had experience as interpreters in a psychotherapy setting may have more previous knowledge and motivation, and may, therefore, be in a position to process the information better. On the other hand, regarding workshop participants, those participants with prior experience as interpreters in a therapy setting may have developed complex attitudes and knowledge about therapy. In the case of an inappropriate attitude, they would have to accept that their unhelpful attitudes were invalid. This is more difficult than forming a new opinion (Bohner and Dickel, 2011).

This study addressed the need for systematically evaluated training programs for interpreters in mental health care services for minor refugees. An online training program for interpreters, who are to translate for minor refugees in the context of TF-CBT in line with the manual by Cohen et al. (2017), was evaluated using pre- and post-tests. Based on the four levels of evaluation (Kirkpatrick, 1975; Kirkpatrick and Kirkpatrick, 2010), the current state of research described above, and clinical experience in the field (Unterhitzenberger et al., 2015, 2019), the study raised the following explorative research questions: Is the interpreter training rated positively by the participants in terms of perceived acceptance and usefulness? Is there a knowledge gain and a shift toward an attitude that is more helpful for therapy over the course of the workshop? In addition, the present study looked at the impact of knowledge gain about TF-CBT, previous experience as an interpreter in general, and prior experience as an interpreter in psychotherapy on changes in attitude.

## Materials and methods

### The workshop

This one-day training program was developed within the framework of the collaborative project “BETTER CARE” (Rosner et al., 2020). It drew on clinical experience in the field and existing best practice approaches (Tribe and Morrissey, 2004; Hanft-Robert et al., 2018), and was piloted by a focus group in November 2019. As an addition, accompanying workshops for therapists were conducted within the scope of BETTER CARE that addressed important considerations when working with interpreters to improve collaboration.

The training sessions were conducted once a month from November 2020 to June 2021 via Zoom video conferencing. In total, there were nine four-hour workshops. The workshops were held on Friday afternoons, apart from two workshops on Monday afternoons. Each workshop was attended by *M* = 14.33 participants (*SD* = 2.05, range 10–17).

The TF-CBT-specific (Cohen et al., 2017) workshop had the following thematic contents: (1) Background information on trauma and PTSD including flight-specific stressors and diagnostics with children and adolescents. (2) A framework for good cooperation and translation in therapy including verbatim translation, non-omission of praise and repetitions in the translation process so as to show comprehension, and translating in the first person singular. In addition, the opportunities to address misunderstandings by talking to the therapist in pre- and post-session (de-)briefings, were explained. Other important issues involved maintaining neutrality (for example, by avoiding private contact with the patient), methods to cope with personal distress, and best-practice seating arrangements. (3) Description of the TF-CBT modules (Cohen et al., 2017). In addition to knowledge transfer through lectures, psychotherapy videos were used to illustrate the therapy modules. (4) Training where the participants had an opportunity to apply their acquired knowledge to case vignettes and exercises. The workshop ended with a discussion in which open questions and further steps in the project could be discussed. Both psychotherapy videos and case vignettes were retrieved from the TF-CBT online learning platform (https://tfkvt.ku.de/) which was developed for German therapists with added sections about culturally sensitive considerations during psychotherapy.

### Recruitment and procedure

**FIGURE 1:**
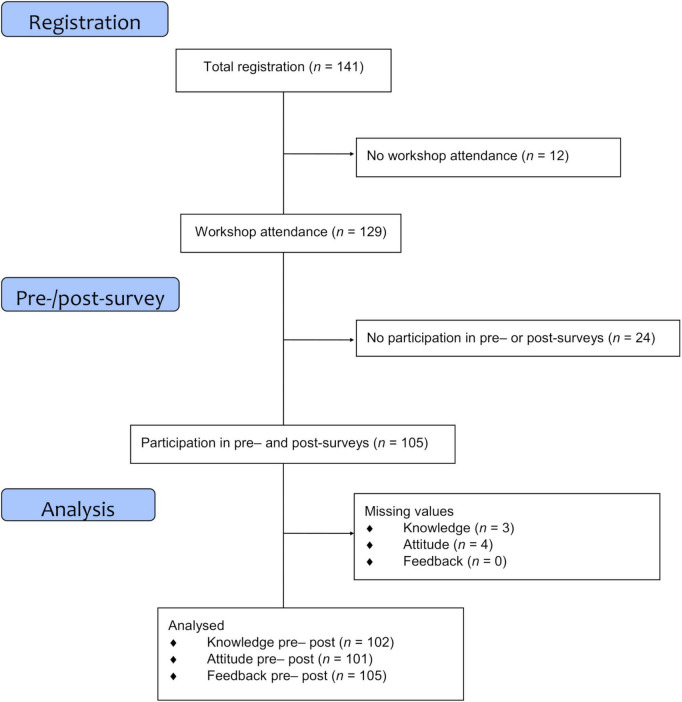
Flow chart of the recruitment process.

The interpreters were recruited through institutions in southern Germany working with community interpreters, contact lists of youth welfare institutions participating in the “BETTER CARE” project and advertisement via Facebook. The workshop was free of charge and the participants in the attached pre- and post-surveys were given a voucher worth 20 EUR. The pre-test was concluded on average 7.65 days (*SD* = 4.09, range 0–16) prior to the workshop and the post-test was completed on average 3.21 days (*SD* = 4.08, range 0–21) after the workshop. To increase completion rates, the participants received two reminder emails and two phone calls if they had not responded to the survey within a week. There were *n* = 141 registrations for the workshop, and *n* = 129 participants actually attended the training course (see Figure 1). A total of *n* = 105 participants completed both the pre- and the post-tests. Datasets were deemed to be complete when they had less than two unanswered items for *n* = 102 participants with regard to the TF-CBT knowledge test and for *n* = 101 with regard to the attitude questionnaire after the workshop had taken place.

### Participants

Table 1 gives the sociodemographic characteristics of the participants. All participants were fluent in German and in at least one other language, and worked as community interpreters. Forty-four (36.07%) males, 70 (57.38%) females and 2 (1.64%) people who gave “diverse” as their gender, participated in the workshop. On average, the participants were 44 years of age (*M* = 43.9, *SD* = 13.6, range 19–87) and had been living in Germany for 19 years (*M* = 19.4, *SD* = 13.2, range 2–56). Twelve (9.84%) of the participants had no professional degree, 36 (29.51%) an apprenticeship certificate, 32 (26.23%) a bachelor’s degree, 27 (22.13%) a master’s degree, 5 (4.10%) a doctorate, and 10 (8.20%) indicated “other”. The group of participants was also very heterogeneous in terms of their religious background [Muslim, *n* = 64 (52.46%), Christian, *n* = 23 (18.85%), non-religious/atheist/agnostic, *n* = 19 (15.57%); Jews, *n* = 2 (1.64%); Hindu, *n* = 1 (0.82%), Yazidi, *n* = 1 (0.82%), other religious background, *n* = 12 (9.84%)].

**TABLE 1:** Sociodemographic characteristics of the participants (*n* = 122) in the pre-tests.

Age in years, *M* (*SD*)	43.9 (13.6)
**Gender, *n* (%)**	
Male	43 (36.07)
Female	77 (75.38)
Diverse	2 (1.64)
**Languages, *n* (%)**	
Arabic	44 (36.07)
Dari	21 (17.21)
French	17 (13.93)
Farsi	15 (12.30)
English	14 (11.48)
Kurdish	14 (11.48)
Turkish	12 (9.84)
Other*	62 (50.82)
**Religion, *n* (%)**	
Muslim	64 (52.64)
Christian	23 (18.85)
Non-religious/atheist/agnostic	19 (15.57)
Jew	2 (1.64)
Hindu	1 (0.82)
Yazidi	1 (0.82)
Other religious background	12 (9.84)
Time working as an interpreter in years, *M (SD)*	6.32 (6.54)
Length of stay in Germany in years, *M* (*SD*)	19.4 (13.20)
**Professional degree, *n* (%)**	
No professional degree	12 (9.84)
Apprenticeship	36 (29.51)
Bachelor’s degree	32 (26.23)
Master’s degree	27 (22.13)
Doctorate	5 (4.10)
Other	10 (8.20)
Qualification or training as an interpreter, *n* (%)	40 (32.79)
**Current field of activity as an interpreter, *n* (%)**	
Migration and asylum counseling	74 (60.65)
Youth welfare services	58 (47.54)
Other health care services	56 (45.90)
Psychosocial services	45 (36.89)
Federal Office for migration and refugees	22 (18.03)
Court	21(17.21)
Translation office	18 (14.75)
Community interpreter	17 (13.93)

*Pashto, Russian, Tigrinya, Sorani, Spanish, Amharic, Somali, Italian, Hindi, Croatian, Mandinka, Portuguese, Vietnamese, Armenian, Bulgarian, Chinese, Greek, Oromiffa, Polish, Slovak, Tajik, Tamil, Telugu, Czech, Chechen, Hungarian, Urdu, Wolof.

Regarding the participants’ experience as interpreters, 40 (32.79%) interpreters stated that they had some form of qualification including attendance of a range of short workshops similar to the present workshop as well as certified diplomas. In contrast, 82 (67.21%) participants did not report having any qualifications as an interpreter at all. On average, the participants had been working as interpreters for 6 years (*M* = 6.32, *SD* = 6.54, range 0–30). The most frequently mentioned fields of activity in which the interpreters were already actively involved, were migration and asylum counseling (*n* = 74, 60.65%). Furthermore, the interpreters already had experience working with the youth welfare services (*n* = 58, 47.54%), other health care services (*n* = 56, 45.90%), and psychosocial services (*n* = 45, 36.89%). Less frequent mention was made of the Federal Office for Migration and Refugees (*n* = 22, 18.03%), courts (*n* = 21, 17.21%), translation offices (*n* = 18, 14.75%), and working as a community interpreter (*n* = 17, 13.93%). Multiple answers were possible for the question about fields of activity.

In total, the interpreters who participated in the workshops covered 35 languages including the languages spoken the most frequently by young refugees (see Table 1). The 3 most common languages were Arabic (*n* = 44, 36.07%), Dari (*n* = 21, 17.21%), and French (*n* = 17, 13.93%).

### Measures

The pre- and post-tests were conducted online using the Qualtrics feedback software (Qualtrics, 2005). Participants were sent the link to the pre-survey one week prior to the training. The link to the post-survey was sent directly after the training program. Participants were asked to complete the post-survey within two weeks of attending the workshop.

#### Sociodemographic survey

The sociodemographic questionnaire consisted of 16 items pertaining to sociodemographic data such as age, origin, religion, profession and education, training as an interpreter, fields of activity and interpreting languages as well as levels of language skills. The items contained open-ended questions and predetermined response options.

#### Training evaluation

To examine participant satisfaction with and the perceived usefulness of the workshop, they completed a questionnaire with 13 items using a five-point Likert scale, ranging from 1 “do not agree at all” to 5 “agree absolutely”. The first eight questions focused on perceived knowledge gain about the therapy and understanding of both the patient’s and therapist’s needs. Furthermore, the first part examined whether the information was helpful for working as an interpreter in psychotherapy in general and in trauma therapy in particular and whether the subject matter was adequate. The following five items included questions about satisfaction with the workshop and perceived appreciation. The survey concluded with an open question that provided an opportunity for further comments. Internal consistency of the total scale was good (ω = 0.96).

#### Psychotherapy perceptions and experiences

Given the lack of established quantitative measures in the field, the psychotherapy perceptions and experiences questionnaire, comprising 15 items, was developed on the basis of the existing literature (Downing and Helms, 1992; Tribe and Morrissey, 2004; Miller et al., 2005; Morina et al., 2010; Gartley and Due, 2017; Hunt and Swartz, 2017) and a consensual expert focus group. The first five questions were part of the pre-survey only. They explored experiences in the field of psychotherapy and trauma therapy. The next two items dealt with the translation of cultural aspects and contained one question in a multiple-choice format and one open question. One subscale of the questionnaire, the Interpreters’ Perceptions and Attitudes in Psychotherapy Scale (IPAP) was relevant for this study. It comprised eight items that measured attitudes toward trauma therapy on a five-point Likert scale ranging from (1) “do not agree at all” to (5) “agree absolutely”. This subscale focused on attitudes toward subjects such as working with the therapist as a team, ensuring clear role division, and verbatim translation during therapy. After inverting the negatively poled items, the sum score ranged from 8 to 30, with higher scores indicating attitudes more helpful for therapy. The internal consistency of the scale was rather low (ω = 0.66).

#### Adapted TF-CBT test

The adapted TF-CBT test (Heck et al., 2015) measured knowledge about PTSD and TF-CBT. It was used to measure knowledge gain over the course of the workshop. The original TF-CBT test for therapists consisted of 40 items (Heck et al., 2015). In this study, we adapted the original version and interpreters answered eight questions relevant to the work of interpreters in TF-CBT before and after the workshop. This modified single choice test contained four items that tested knowledge about the symptoms and causes of PTSD and its diagnosis as well as four items focusing on empirical evidence, duration, and the main concepts of TF-CBT (ω = 0.61).

#### Data analysis

Participant satisfaction and perceived acceptance were analyzed descriptively using a histogram, and by running a mean and variance value calculation of the feedback given by the participants. When less than two items in a questionnaire were unanswered, those individual items were calculated using the method “pre for post” or “post for pre” so as to obtain conservative data imputation in all questionnaires. For the calculation of internal consistency of the measures in use, we employed the R package “PSYCH” which allows calculating McDonald’s omega total.

To analyze the data, we relied on the theoretical background of Bayesian estimation according to Kruschke (2011, 2013, 2021). This method has the advantage of permitting statements about probability distributions of the parameter, and not only about the point estimates used in frequentist statistics. Thus, the decision criterion is intuitive and gives information about the uncertainty of the parameter estimations. The highest density interval (HDI) has a similar function to the confidence interval in frequentist statistics. It is defined as the 95% highest density of the posterior distribution. The null hypothesis is rejected if the HDI does not contain the null. Even if the 95% is as randomly chosen as the *p*-value in frequentist statistics, the HDI can be interpreted much more easily, and provides more information. A wide HDI range means a high degree of uncertainty, an indication that the range of credible parameters is wide. A small HDI, on the other hand, indicates a low level of uncertainty, ensuring trust in the credible parameters. Even in this simple procedure of comparing pre- and post-knowledge and attitudes, the Bayesian method furnishes far more information than a simple *t*-test. In addition, statements can be made about mean differences, standard deviations, and effect sizes in one test. Consequently, the estimations are more precise (Kruschke, 2011; McElreath, 2016; Tschirk, 2019). In general, Bayesian statistics, therefore, includes frequentist approaches but also shows how the data would look after resampling as well (McElreath, 2016).

To calculate changes in attitude and knowledge gain, the “BEST” packages as described by Kruschke (2013) in R Core Team (2020) with RStudio Team (2020) were used. While the traditional *t*-test uses normal distribution to describe the data, BEST represents the data by using a larger tailed *t*-distribution (Kruschke, 2013). It includes outliers as well as the densest area of the data. Since there is little reliable data in this field, the vague prior default in the package was not changed. The model is, therefore, dominated by the data and prior knowledge has little impact (Bååth, 2014; Kruschke, 2015). A Marcov Chain Monte Carlo (MCMC) algorithm was used to calculate credible parameter values given the data. MCMC uses the heap of the product of the prior and likelihood function to estimate representative values of parameters and calculate the posterior distribution by generating random samples of those values. Thus, it enables estimations of group differences without relying on mean and standard deviation, by only using representative values of the sample. The algorithm moves through the representative values choosing the next data point, with equal probability, of all adjacent data points. If the value of the chosen adjacent data point is larger than the present one, the algorithm shifts. If it is smaller, the algorithm shifts with the probability of the relation of the chosen adjacent data point’s value to the present data point’s value. By moving through the heap, the algorithm creates the density of credible parameter values of the sample (Kruschke, 2011, 2013, 2015; McElreath, 2016). This procedure enables “BEST” to calculate, for paired samples, the posterior distribution of the paired mean difference, the effect size, and the standard deviation of the paired difference in one test.

To examine the impact of experience as an interpreter and knowledge gain on changes in attitude, the R scripts of Kruschke (2021) “Jags-Ymet-XmetMulti-Mrobust.R” sourced from Jags-Ymet-XmetMulti-Mrobust-Example.R were used. In these scripts, the MCMC was calculated using the JAGS software program after standardization of the data. This script included generating a correlation matrix and visualization of the data. The vague prior in the scripts and the burn in period of 100 of the MCMC were left unchanged. The Breusch-Pagan Test was used to verify the assumed homogeneity of variance.

## Results

### Feedback, knowledge, and attitude change

**FIGURE 2:**
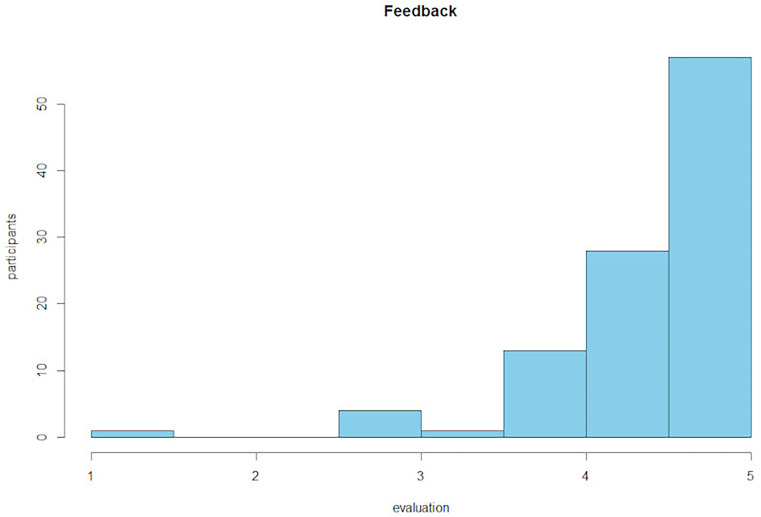
Histogram of participants’ average rating of the training evaluation questionnaire.

Figure 2 gives the participants’ average rating of the feedback on perceived usefulness and acceptance of the workshop (*M* = 4.45, *SD* = 0.64).

**FIGURE 3:**
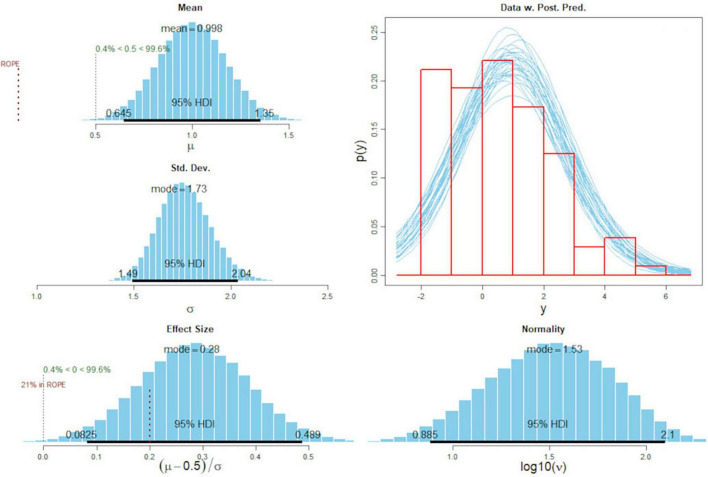
Posterior distribution of mean difference and effect size of right answers in the adapted TF-CBT test pre- and post-surveys. Standard deviation of differences, normality, and post predictive check.

A total of *n* = 102 participants completed the adapted TF-CBT knowledge test in both the pre- and post-surveys. Figure 3 gives the posterior distribution of the mean differences in the pre- and post-survey, the effect size, the standard deviation of differences, and the posterior predictive check. The MCMC used by “BEST” (Kruschke, 2013) calculated a knowledge gain over the course of the workshop with a mean difference of 0.998, as shown in the upper left graphic (credible interval 0.65–1.35). The posterior distribution of the effect size yielded a median of 0.28 and a HDI of between 0.083 and 0.49. The posterior predictive check, representing the possible probability functions as presented in the lower right graphic, showed a model fit that represented the slightly skewed data relatively well. In sum, the Bayesian estimation indicated a mean difference of more than 0 with a probability of > 99.6%.

**FIGURE 4:**
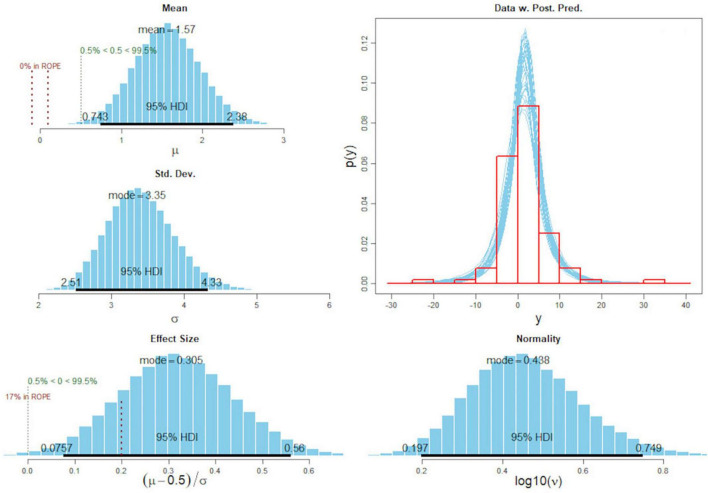
Posterior distribution of mean difference and effect size of the IPAP pre- and post-survey sum scores. Standard deviation of differences, normality, and post-predictive check.

A total of *n* = 101 participants completed the IPAP in both the pre- and the post-surveys. The MCMC simulation with “BEST” revealed a median of the mean paired difference of 1.57 with a HDI of between 0.74 and 2.4 (see Figure 4). The effect size resulted in a median of 0.31 and a credible interval between 0.08 and 0.56. The posterior predictive check, representing the possible probability functions as shown in the lower right graphic, pointed to a good model fit. In sum, the Bayesian estimation indicated a mean difference of more than 0 with a probability of 99.5%.

### Predictors of attitude change

**FIGURE 5:**
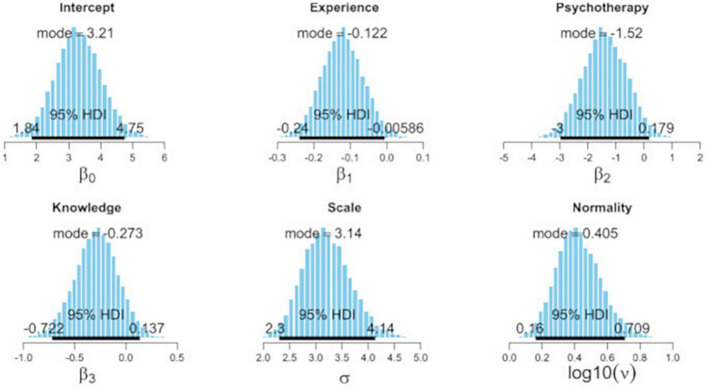
Highest density interval (HDI) of regression coefficients: experience in years, experience in psychotherapy, and knowledge gain.

Figure 5 gives the posterior distribution and HDI of the intercept as well as the posterior distribution and HDI of the slope values of the regression coefficients that generate the regression function. Critical values for the intercept (mode = 3.21, HDI = 1.84–4.75), attitude change conditioned by experience (mode = −0.12, HDI = −0.24 to −0.006), experience in psychotherapy (mode = −1.52, HDI = −3.00 to 0.18), and knowledge gain (mode = −0.27, HDI = −0.72 to 0.14) were estimated. The posterior distribution of the slope value of experience in psychotherapy and knowledge gain included the null but suggested a negative trend. However, the HDI of the slope value of the impact of experience as an interpreter in years on changes in attitude change did not include the null, indicating a negative impact of professional experience on attitude change. The proportion of variance accounted for by the model, the equivalent to R^2^ in traditional least square multiple regression, indicated mode = 0.053, HDI = 0.01–0.09.

## Discussion

In this study we reported on the evaluation of a TF-CBT-specific workshop for interpreters working in mental health care for minor refugees. To the authors’ knowledge, it was the first quantitative evaluation of an interpreter training program specific to collaboration in psychotherapy. It, therefore, constitutes pioneer work in the field of mental health care for children and adolescents. The demographical background of the participants was very heterogeneous in terms of age, experience, and professional qualifications. Although 80% of the participants had at least completed an apprenticeship or reported higher levels of education, two-thirds of the interpreters working mostly in the fields of migration and asylum counseling, youth welfare services, health care or psychosocial services in Germany, did not report having any qualifications as interpreters in this professional area. Various risk factors come into play when employing untrained interpreters (Paone and Malott, 2008; Crezee et al., 2013). Interpreting for minors requires especially skillful and high-quality translation (Raval, 2003; Rousseau et al., 2011). This re-emphasizes the importance of scientifically evaluated workshops and training programs for interpreters to ensure access to and dissemination of evidence-based therapies. This has been pointed out by a variety of authors (Cecchet and Calabrese, 2011; Searight and Armock, 2013; Hunt and Swartz, 2017; van Os et al., 2020). Given the paucity of these evaluated training programs, the results of this study on a TF-CBT-specific workshop are promising.

Despite the heterogeneity of the participants, the results of this study indicated that there had been an increase in trauma- and TF-CBT-related knowledge and attitudes considered beneficial for working as an interpreter in psychotherapy over the course of the workshop (Paone and Malott, 2008). The knowledge gain was around one point (with a rather low effect size) indicating that, on average, the participants answered one more question correctly after the workshop than in the pre-workshop test. As there were only eight questions and the knowledge level was already high before the workshop, this knowledge gain can be deemed a success. Interpreters working in a psychotherapy setting are more than a mere mechanical medium for translation and are, as persons, an integral part of the procedure (Schouler-Ocak and Aichberger, 2017), especially when working with minors. They, therefore, need to grasp the basic concepts of TF-CBT methods and the special characteristics of the clinical picture of PTSD (Cecchet and Calabrese, 2011). Even assuming there is clear role clarification, it might be much easier for trained interpreters to translate repetitions, praise and every word of the patient, if they are aware of the importance of these aspects for successfully delivering TF-CBT (Paone and Malott, 2008; Butow et al., 2012). The knowledge gain at the workshop may, therefore, lead to improved teamwork between the therapist and the interpreter as both have a similar understanding of psychotherapy and know how TF-CBT works.

In terms of attitude change, an increase in attitudes beneficial to psychotherapy was observed with a high degree of probability. As the distribution of attitudes beneficial to therapy was already skewed toward high levels of attitudes beneficial to therapy at the time of pre-testing, ceiling effects were possible (Döring and Bortz, 2016). Given the short duration of four hours and the simple and economic didactic structure of the workshop, the results are highly encouraging. This workshop, therefore, took up the challenges described by various authors working in psychotherapy with interpreters (Miller et al., 2005; Morina et al., 2010; Gartley and Due, 2017; Kießl et al., 2017; Hanft-Robert et al., 2018) by addressing problems such as role confusion (Morina et al., 2010) and different translating norms (Morina et al., 2010; Kießl et al., 2017; Hanft-Robert et al., 2018), and by discussing them with the interpreters. Consequently, the expectations of the interpreter’s role developed in the workshop provided interpreters with clear guidelines that could facilitate cooperation between them and the therapist. This might not only improve the quality of therapy but also protect them from the psychosocial consequences of role confusion (Teegen and Gönnenwein, 2002; Butow et al., 2012). The importance of such guidelines was reflected in discussions during the workshop, in which the question arose very frequently as to how a clear division of roles could be maintained and how it would be possible to distance oneself from the patient’s concerns.

The positive findings concerning the effect of the online training for interpreters specific to TF-CBT were also underpinned by the very positive feedback given by the participants as seen in the data and also verbally. Regarding the positive feedback, it is important to note that participants did not have to pay for the workshop which might have led to socially desirable response patterns.

When looking at Kirkpatrick’s four levels of evaluation (Kirkpatrick, 1975; Kirkpatrick and Kirkpatrick, 2010), we could see that the participants reacted very positively to the training (reaction) and that knowledge increased and attitude changed over the course of the workshop (learning). The next two steps according to Kirkpatrick (1975) would be to investigate whether the participants changed not only their attitudes but also their behavior (behavior) and if therapy with trained interpreters was more effective (results). Promising results of short educational workshops on other psychosocial subjects suggest that such workshops may have an impact not only on knowledge gain and attitude change, but also on behavior change (Sawyer et al., 2016; Marcussen et al., 2019; Pontes et al., 2019). In these studies, based on Kirkpatrick’s levels of evaluation, hospital staff demonstrated more positive attitudes toward collaboration with patients, improved role clarity and individual authority, the desired behavior change, and patient satisfaction. Given the promising results for knowledge gain and attitude change in the present study, similar trends could, therefore, be possible for this interpreter-specific training with regard to behavior change and more qualified psychotherapy when working with trained interpreters in mental health care for minor refugees.

The predictors “experience in therapy” and “knowledge gain” showed tendencies of negative effects on attitude change. This is in line with earlier findings among other professions (Chaiken and Maheswaran, 1994; Dillard and Pfau, 2002; Kruglanski et al., 2006; Bohner and Dickel, 2011; van Lange et al., 2011). The predictor “experience in therapy” was negatively associated with attitude changes with more than 95% probability. This indicated that participants with more experience as interpreters were less likely to change their attitudes. According to the literature, this could be due to existing entrenched attitudes (Bohner and Dickel, 2011) biased by previous information (van Lange et al., 2011). As the workshop was run by a university member, the participants could have changed their attitude simply because they had confidence in the “scientific” speaker (peripheric process) (Chaiken and Maheswaran, 1994; van Lange et al., 2011). This was particularly true for participants with no prior experiences of trauma therapy, as they had less reference knowledge that would have enabled them to challenge the information provided. Furthermore, changing attitudes about working as an interpreter when already working as an interpreter in psychotherapy included the critical questioning of one’s own work. This might lead to a more self-protecting and defense-motivated position (van Lange et al., 2011). Moreover, it seemed likely that experienced interpreters already had desired attitudes that were probably caused by ceiling effects and were less likely to change.

### Strengths and limitations

To the author’s knowledge, the present study is one of the first to systematically evaluate a training program for interpreters working in mental health services and trauma-focused psychotherapy and, by extension, to address the lack of qualified training (Cecchet and Calabrese, 2011; Searight and Armock, 2013; Hunt and Swartz, 2017; van Os et al., 2020) and its evaluation (Rousseau et al., 2011) specific to minors (Raval, 2003; Rousseau et al., 2011). The present study, therefore, looked at the scarcity of existing research and this was even more necessary with regard to a therapeutic setting in a triad with minor patients. Using Bayesian statistics in this exploratory approach offered the advantage of being able to calculate probabilities and uncertainties and, by extension, to obtain more information about the different parameters. In addition, as Bayesian statistics take existing scientific knowledge into account, this method will enable more extensive research in the context of interpreter workshops on attitude and knowledge changes to enable the use of our outcomes as new prior knowledge.

Nevertheless, it must be emphasized that this study adopted an exploratory approach. There was no comparison training or control sample consisting of untrained individuals. In addition, most of the instruments used had not yet been validated. Consequently, no information was available on how effectively the constructs had been measured. Furthermore, the questionnaires did not permit any comparison with the average population. This is due, by and large, to the lack of established measures in the context of attitude change among interpreters in mental health settings. The internal consistency of the IPAP was rather low which might indicate that it had captured divergent constructs. This is a major limitation of the study and further research is needed to develop psychometrically validated questionnaires to capture helpful attitudes of interpreters for therapy. Moreover, there was no exploration of whether the workshop had increased the quality of translation in therapy and, by extension, the effectiveness of therapy since no external control of success, such as the comparison of therapy results of trained and untrained interpreters, was carried out. Even if the results of the present study constituted important initial pointers in a field in which hardly any findings exist, they should still be interpreted cautiously.

### Implications and conclusion

The promising results emphasize the importance and effectiveness of simple and cost-effective workshops for interpreters working in (trauma-focused) psychotherapy. In order to improve training programs for interpreters involved in psychotherapy, there is still need for validation of instruments screening for therapy-relevant attitudes. Moreover, the results of trained interpreters should be compared with control groups. Further research is essential in order to provide insight into the effectiveness of training in terms of behavior change, and to ensure qualitative therapy with trained interpreters. Additionally, more workshops for interpreters focusing on work in psychotherapy need to be developed and evaluated scientifically. As psychotherapy in the presence of an interpreter was shown to be effective provided the interpreters were experienced or educated (Ardenne et al., 2007; Brune et al., 2011; Lambert and Alhassoon, 2015), evaluated training programs on trauma therapy for interpreters are an important basis for appropriate therapeutic healthcare when therapist and patient do not speak the same language. Based on this, quality standards for training could be put in place and disseminated (Cecchet and Calabrese, 2011; Searight and Armock, 2013; Hunt and Swartz, 2017; van Os et al., 2020). This could enable therapists and patients to find and identify well-trained interpreters in the language needed. Stepped-care approaches such as the BETTER CARE project (Rosner et al., 2020), to which the present study was linked, offer a good framework for the further dissemination of interpreter training specific to trauma therapy.

## Data availability statement

The raw data supporting the conclusions of this article will be made available by the authors, without undue reservation.

## Ethics statement

The studies involving human participants were reviewed and approved by Institutional Review Board of the Catholic University Eichstätt-Ingolstadt. The patients/participants provided their written informed consent to participate in this study.

## Author contributions

LM developed and conducted the workshops and supervised the study process. MH helped to conduct the workshops, collected the data, carried out the data analysis, and drafted the manuscript. JU co-designed the workshop and supervised the study process. RR was the principal investigator of the study and supervised the study process. All authors read and approved the final manuscript.
